# Vulnerability to traumatic stress in fibromyalgia patients: 19 month follow-up after the great East Japan disaster

**DOI:** 10.1186/ar4310

**Published:** 2013-09-23

**Authors:** Chie Usui, Kotaro Hatta, Satoko Aratani, Naoko Yagishita, Kenya Nishioka, Satoko Okamura, Kenji Itoh, Yoshihisa Yamano, Hiroyuki Nakamura, Nozomu Asukai, Toshihiro Nakajima, Kusuki Nishioka

**Affiliations:** 1 Department of Psychiatry, Juntendo University Nerima Hospital, 3-1-10 Takanodai, Nerima-Ku, Tokyo 177-8521, Japan; 2 Institute of Innovative Medical Science and Education, Tokyo Medical University, 6-1-1 Shinjyuku, Shinjyuku-ku, Tokyo 160-8402, Japan; 3 Institute of Medical Science, St. Marianna University School of Medicine, 2-16-1 Sugo, Kawasaki, Miyame-ku, Kanagawa 216-8511, Japan; 4 Department of Neurology, Juntendo University School of Medicine, 2-1-1 Hongo, Bunkyo-Ku, Tokyo 113-8421, Japan; 5 Kochi-Daiichi Hospital, 2-14 Kutanda, Kochi 780-0832, Japan; 6 Department of Rheumatology, National Center for Global Health and Medicine, 1-21-1Toyama, Shinjuku-ku, Tokyo 162-8655, Japan; 7 Department of Environmental and Preventive Medicine, Graduate School of Medical Science, Kanazawa University, Kakuma-cho, Kanazawa-city, Kanazawa 920-1192, Japan; 8 Department of Psychiatry and Behavioral Sciences, Tokyo Metropolitan Institute of Medical Science, 2-1-8 Kamikitazawa, Setagaya-ku, Tokyo 156-8506, Japan

## Abstract

**Introduction:**

The aim of this study was to investigate vulnerability and long-term influence of traumatic stress caused by the Great East Japan Disaster which occurred on March 11, 2011, in patients with fibromyalgia, which is a chronic pain syndrome probably involving central sensitization.

**Methods:**

A total of 60 female patients with fibromyalgia were compared with female patients with rheumatoid arthritis (RA, n = 23) as another chronic pain disease, and with female healthy controls (HC, n = 26) in the observational study. To evaluate responses to traumatic stress, the scores of Impact of Event Scale-Revised (IES-R) were assessed one month after the disaster and every six months until 19 months after the disaster. We also evaluated levels of depression during the study period. To know the score of IES-R of patients with fibromyalgia during usual living, we assessed IES-R in another population of fibromyalgia patients without exposure to a great disaster.

**Results:**

The mean score of IES-R one month after the disaster in the fibromyalgia group (24.6 [SD 18.9]) was significantly higher than that of RA group (13.4 [SD 14.5]) or HC group (9.1 [9.2]) (F = 9.96, p < 0.0001). However, the mean score of IES-R in fibromyalgia patients without exposure to a great disaster was (20.3 [SD 18.7]), which was almost the same value as the fibromyalgia group seven months after the disaster (20.2 [SD 19.5]). Repeated measures analysis of variance showed significant effect of time course in the depression-related symptoms (F = 6.68, P = 0.001), and a post-hoc test revealed that the number of depression-related symptoms one month before the disaster was significantly different from other time points until 19 months after the disaster, respectively.

**Conclusions:**

Although response to acute stress induced by the great earthquake was likely to be settled within seven months after the disaster, depression-related symptoms have been increasing for more than one year after the disaster, despite exclusion of patients with major depression at baseline. This long-lasting worsening of depression-related symptoms may have been in response to chronic stress induced by the fear of radiation due to the nuclear power disaster. These findings suggest that patients with fibromyalgia are vulnerable to chronic stress rather than acute stress.

## Introduction

On 11 March 2011, a magnitude 9.0 earthquake struck the east coast of Japan. The total number of people who died in the earthquake and the subsequent tsunami is approximately 19,000. To make the situation worse, the Fukushima nuclear power plants were seriously damaged and it took 9 months to settle the subsequent nuclear reactor problems. The earthquake registered 5 to 6 on the Japan Meteorological Agency seismic intensity scale in Tokyo areas, which caused acute stress to people who lived in Tokyo. The nuclear power disaster caused continuous stress not only to people in the disaster-stricken areas but also to people in Tokyo areas due to records above the normal range of radiation during 1 month after the disaster (Tokyo Metropolitan Institute of Public Health). The response to such acute and subacute traumatic stress should therefore have been monitored even in Tokyo, especially in patients with diseases involving psychological factors.

Fibromyalgia is characterized by widespread musculoskeletal chronic pain, fatigue, poor sleep, frequent psychological difficulties, and multiple tender points on physical examination [1,2]. The etiology of fibromyalgia is unknown but may involve neuropsychiatric vulnerability [3]. There is evidence of the relationship between traumatic experiences and prevalence of fibromyalgia diagnosis [4,5], but a long-term follow-up study does not exist. Meanwhile, effects of traumatic stress induced by the World Trade Center terrorist attacks on pain have not been detected between before and after 11 September 2001 [5].

The aim of this study was to investigate vulnerability and long-term influence to traumatic stress caused by the Great East Japan Disaster in patients with fibromyalgia, compared with patients with rheumatoid arthritis (RA) as another chronic pain disease and with healthy controls. In addition, change in the severity of fibromyalgia between before and after the disaster was also examined.

## Methods

We recruited fibromyalgia patients who had been followed up before the earthquake in a clinic specialized for fibromyalgia, the Kasumigaseki Urban Clinic, in Tokyo, Japan, between 11 April and 18 April 2011. Fibromyalgia was diagnosed according to the previous criteria for the 1990 classification of the American College of Rheumatology [1]. Patients concomitant with psychiatric disorders according to the Diagnostic and Statistical Manual of Mental Disorders – IV [6] were excluded. Also, patients whose prescription had been changed at their visit to the clinic just before the disaster were excluded. During the study period, RA patients who had been followed up before the disaster were also recruited to represent another musculoskeletal chronic pain disease. The diagnosis of RA was made according to the 2010 RA classification criteria [7]. Healthy subjects from hospital workers were also enrolled as controls. All subjects in the present study neither received direct physical harm nor were exposed to harmful level of radiation. Also they did not lose their family member lives. This study was approved by the Institutional Review Board of Kasumigaseki Urban Clinic. Written informed consent was obtained from all participants.

To evaluate responses to traumatic stress, scores on the Impact of Event Scale – Revised (IES-R) were assessed 1 month after the disaster and every 6 months until 19 months after the disaster in the three groups: fibromyalgia patients, RA patients, and healthy controls. The IES-R is a 22-item self-rating scale to evaluate traumatic stress symptoms developed by Weiss [8]. The scale consists of three subscales: Intrusion, Avoidance, and Hyperarousal. The Japanese-language version of the IES-R has been well validated [9].

The severity of fibromyalgia was assessed using the Fibromyalgia Symptom Scale (mFS-J [10]). The mFS-J consists of the Widespread Pain Index (WPI) and the Symptom Severity scale. The WPI represents the number of areas in which the patient has had pain over the last week. The rater examines whether patients have pain or not in 19 areas of their body. The score will be between 0 and 19. The Symptom Severity scale is the sum of the severity of the three symptoms (fatigue, waking unrefreshed, cognitive symptoms) plus the extent (severity) of somatic symptoms in general. The final score is between 0 and 12. At the clinic, fibromyalgia patients had been assessed by the first and last authors using the mFS-J at every visit before the disaster. The intra-class correlation coefficient between the two independent raters was very high for the mFS-J, at 0.994 [10]. Pre-disaster scores of the mFS-J in the present study were based on the assessment records within 1 month before the disaster. The mFS-J was then assessed by the first author 1 month after the disaster and every 6 months until 19 months after the disaster.

Similarly, the severity of RA after the disaster was compared with that before the disaster according to inflammation data of RA, such as C-reactive protein, white blood cell counts, and the erythrocyte sedimentation rate. The pre-disaster data had been collected within 1 month before the disaster.

To evaluate levels of depression during the study period, we identified five depression-related symptoms common to mFS-J and a major depressive episode of the Diagnostic and Statistical Manual of Mental Disorders – IV (that is, fatigue/tiredness, thinking of or remembering problems, insomnia, depression, and loss of appetite), and analyzed the time course of the total number of these symptoms present.

To determine the IES-R score for fibromyalgia patients during usual living, we recruited age-matched patients with fibromyalgia who were outpatients of Daiichi Hospital located in Kochi-City, in the western part of Japan, in June 2013. Inclusion criteria were being female and without exposure to a great disaster. At the same time, we assessed the IES-R for fibromyalgia patients in Tokyo we followed up.

### Statistical analysis

Data were analyzed using SPSS 20-J software (IBM Japan, Tokyo, Japan). Differences among groups in demographic and clinical characteristics were calculated with one-way analysis of variance (ANOVA) and the *post-hoc* Tukey–Kramer multiple comparisons test was used. If data were not sampled from Gaussian distributions, a nonparametric test (Mann–Whitney *U* test) was used. To compare categorical data, we used Fisher’s exact test. To examine changes in the score for mFS-J or depression-related symptoms, repeated-measures ANOVA was used. We compared the IES-R scores in subjects among fibromyalgia, RA patients, and controls after correcting for age, using one-way analysis of covariance (ANCOVA) with age as covariate. For multiple comparisons, the Bonferroni test was used. Linear correlation between IES-R scores and mFS-J and its subscale scores was examined. All statistical tests were two-tailed. Statistical significance was set at *P* <0.05.

## Results

### Baseline characteristics

A total of 80 female patients with fibromyalgia, 32 female patients with RA, and 30 female healthy controls were enrolled, and 60 (75%), 23 (72%), and 26 (87%) subjects completed the follow-up period, respectively. Other patients dropped out due to a change of clinics, and four healthy controls dropped out due to a change of jobs. Demographic and clinical characteristics of the groups are presented in Table 1.

**Table 1:** Baseline characteristics of patients

	**Healthy control**	**Rheumatoid arthritis**	**Fibromyalgia**	***P*** **value**
	**(*N *** **= 26)**	**(*N *** **= 23)**	**(*N *** **= 60)**	
Age (years)^a^	42.3 (12.5)	56.0 (14.4)	48.4 (14.8)	0.0047
Asian	26/26 (100)	23/23 (100)	60/60 (100)	
Duration of illness (years)		7.4 (6.7)	6.4 (7.4)	0.31
Living alone^b^	5/26 (19)	3/23 (13)	6/60 (10)	0.32
Unemployed^b^	0/26 (0)	3/23 (13)	9/60 (15)	0.066
Comorbid disease		14/23 (61)	39/60 (65)	0.80

Data represent mean (standard deviation) or *n*/*N* (%), unless otherwise indicated. ^a^Tukey–Kramer multiple comparisons test showed that mean age in the rheumatoid arthritis group was higher than that in the healthy control group (*P* <0.01). ^b^Healthy control group versus others.

### Change in main symptoms in patients with fibromyalgia and patients with RA before and after the disaster

**Figure 1:**
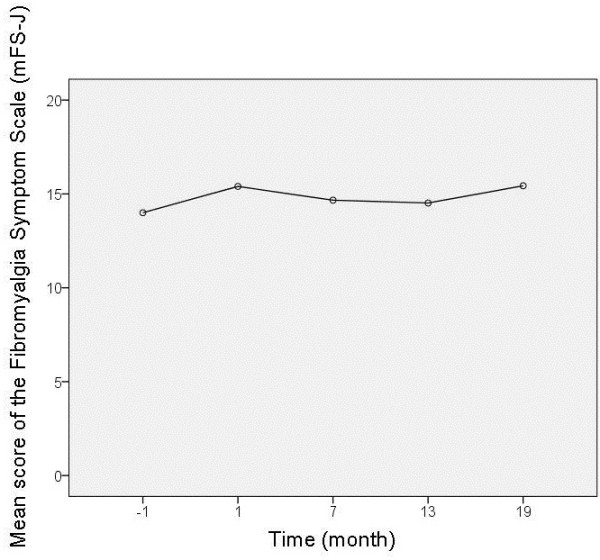
**Change in the score of the Fibromyalgia Symptom Scale.** Repeated-measures analysis of variance revealed no significant effect of time course (*F* = 1.80, *P* = 0.13). mFS-J, Fibromyalgia Symptom Scale.

The change in mFS-J is shown in Figure 1. Repeated-measures ANOVA revealed no significant effect of time course (*F* = 1.80, *P* = 0.13). We also analyzed the time course of the WPI, a score that represents only pain and accounts for 61% (19/31) of the mFS-J, resulting in no significant effect of time course (*F* = 1.30, *P* = 0.28). Similarly, in patients with RA there was no significant change in the mean levels of C-reactive protein, white blood cell counts, and erythrocyte sedimentation rate. Patients with fibromyalgia received the following medication before the disaster: pregabalin, 53 patients; gabapentin, 24 patients; and duloxetine, 12 patients. Patients with RA received the following medication before the disaster: methotrexate, 14 patients; TNFα inhibitors, 13 patients; IL-6 inhibitor, four patients; T-cell activation inhibitor, three patients; and prednisolone, 18 patients. Additional medications including an increase in dose within 6 months after the disaster were 31 for the fibromyalgia group (duloxetine, six patients; benzodiazepines, six patients; gabapentin, five patients; pregabalin, four patients; nonsteroidal anti-inflammatory drugs, four patients; lamotrigine, three patients; quetiapine, three patients) and eight for the RA group (pregabalin, three patients; TNFα inhibitors, two patients; gabapentin, one patient; benzodiazepines, one patient; lamotrigine, one patient). There was no significant difference in the rate between the groups (52% vs. 35%, *P* = 0.22), suggesting only minimal effects of additional medication after the disaster on the present results.

### Development of stress-related symptoms in patients with fibromyalgia, patients with RA, and healthy controls after the disaster

**Figure 2:**
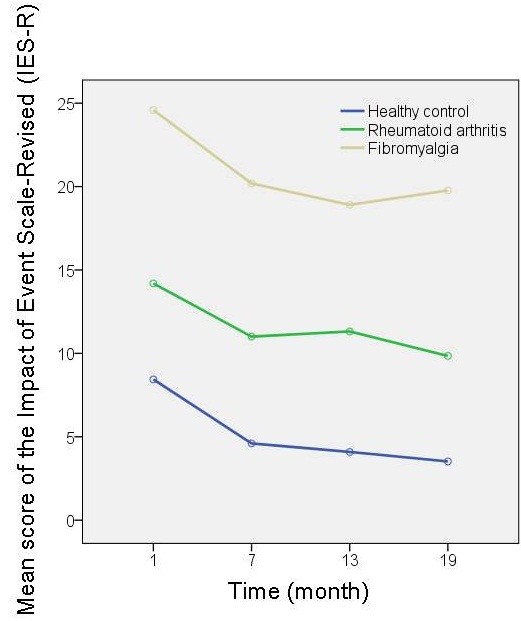
**Change in the score of the Impact of Event Scale – Revised.** Repeated-measures analysis of covariance revealed significant main effects of time course (*F*(3,315) = 3.32, *P* = 0.030) and group (*F*(2,105) = 11.5, *P* <0.0001) on change in the Impact of Event Scale – Revised (IES-R) score. However, there was no significant interaction between time course and group on change in the IES-R score (*F*(6,315) = 0.20, *P* = 0.96).

**Figure 3:**
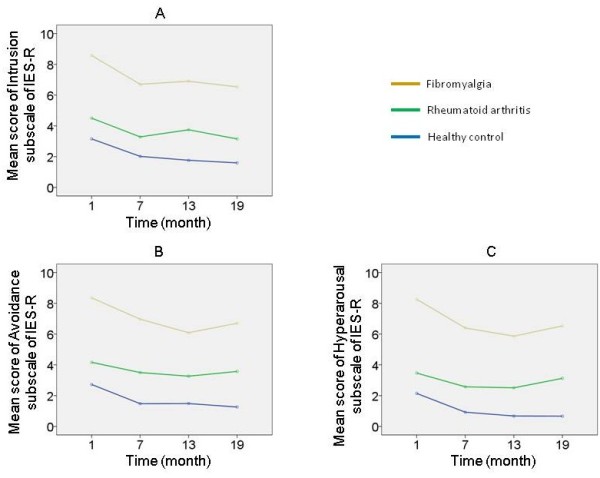
**Change in the subscale scores of the Impact of Event Scale – Revised. (A)** Intrusion: repeated-measures analysis of covariance (ANCOVA) revealed significant main effects of time course (*F*(3,315) = 3.11, *P* = 0.027) and group (*F*(2,105) = 10.1, *P* <0.0001) on change in the Impact of Event Scale – Revised (IES-R) score. However, there was no significant interaction between time course and group on change in the IES-R score (*F*(6,315) = 0.17, *P* = 0.99). **(B)** Avoidance: repeated-measures ANCOVA revealed significant main effects of time course (*F*(3,315) = 4.60, *P* = 0.004) and group (*F*(2,105) = 10.3, *P* <0.0001) on change in the IES-R score. However, there was no significant interaction between time course and group on change in the IES-R score (*F*(6,315) = 0.28, *P* = 0.95). **(C)** Hyperarousal: repeated-measures ANCOVA revealed main effects of time course as trend level (*F*(3,315) = 2.39, *P* = 0.069) and significant main effects of group (*F*(2,105) = 19.2, *P* <0.0001) on change in the IES-R score. However, there was no significant interaction between time course and group on change in the IES-R score (*F*(6,315) = 0.65, *P* = 0.69).

ANCOVA showed that there was significant difference in the mean IES-R score 1 month after the disaster among the three groups after adjusting for age (*F* = 9.96, *P* <0.0001). The Bonferroni *post-hoc* multiple comparisons test showed that the mean IES-R score 1 month after the disaster in the fibromyalgia group (24.6 (standard deviation (SD) 18.9)) was significantly higher than that of the RA group (13.4 (14.5)) (*P* = 0.036) or the healthy control group (9.1 (9.2)) (*P* <0.0001). Repeated-measures ANCOVA revealed significant main effects of time course (*F*(3,315) = 3.32, *P* = 0.030) and group (*F*(2,105) = 11.5, *P* <0.0001) on change in the IES-R score (Figure 2). However, there was no significant interaction between time course and group on change in the IES-R score (*F*(6,315) = 0.20, *P* = 0.96). Similar results were observed in all of three subscales of the IES-R: Intrusion, Avoidance, and Hyperarousal (Figure 3).

There were significant correlations not only between IES-R and mFS-J (*r* = 0.52, *P* <0.0001), but also between IES-R and WPI (*r* = 0.40, *P* = 0.0016) 1 month after the disaster. Also, there were significant correlations at all other following periods between IES-R/mFS-J and IES-R/WPI: *r* = 0.50, *P* <0.0001 and *r* = 0.40, *P* = 0.0017, respectively, for 7 months after the disaster; *r* = 0.51, *P* <0.0001 and *r* = 0.43, *P* = 0.0006, respectively, for 13 months after the disaster; and *r* = 0.51, *P* <0.0001 and *r* = 0.43, *P* = 0.0007, respectively, for 19 months after the disaster. Moderate correlations between the IES-R score and the mFS-J score and between the IES-R score and the WPI score were thus found at time points after the disaster, and fluctuations in the correlations were very small.

### Estimation of the IES-R score in patients with fibromyalgia during usual living

Twenty age-matched patients with fibromyalgia in Kochi were included as another population of fibromyalgia patients without exposure to a great disaster. Meanwhile, among 60 patients with fibromyalgia in Tokyo we followed up, we could assess the IES-R in 58 patients at the same time. The mean age of fibromyalgia patients in Kochi was 50.6 years (SD 12.7), which was similar to the mean age of the fibromyalgia group in Tokyo (50.5, SD 14.3, *t* = 0.024, *P* = 0.98). The mean IES-R score of the fibromyalgia patients in Kochi in June 2013 was 20.3 (SD 18.7), and that of the fibromyalgia patients in Tokyo at the same time was 18.6 (SD 19.4). There was no significant difference in the mean IES-R score between the fibromyalgia patients in Kochi and the fibromyalgia patients in Tokyo (*t* = 0.33, *P* = 0.74), suggesting that the IES-R score of fibromyalgia patients during usual living is approximately 20. Furthermore, the mean IES-R score in fibromyalgia patients without exposure to a great disaster (20.3, SD 18.7) was almost the same value as the fibromyalgia group 7 months after the disaster (20.2, SD 19.5).

These findings strongly suggest that the mean IES-R score 7 months after the disaster in the fibromyalgia group had already returned to baseline. Change in the mean IES-R score between 1 month and 7 months after the disaster was therefore compared between the groups. ANCOVA showed that there was no significant difference in the mean change in score on IES-R among the three groups after adjusting for age (3.8 for healthy controls, 3.2 for RA, 4.4 for fibromyalgia at the age of 48.6; *F* = 0.072, *P* = 0.93). The amplitude of acute response to the event in fibromyalgia patients may thus not necessarily be greater than that of another population.

### Depression-related symptoms in patients with fibromyalgia before and after the disaster

**Figure 4:**
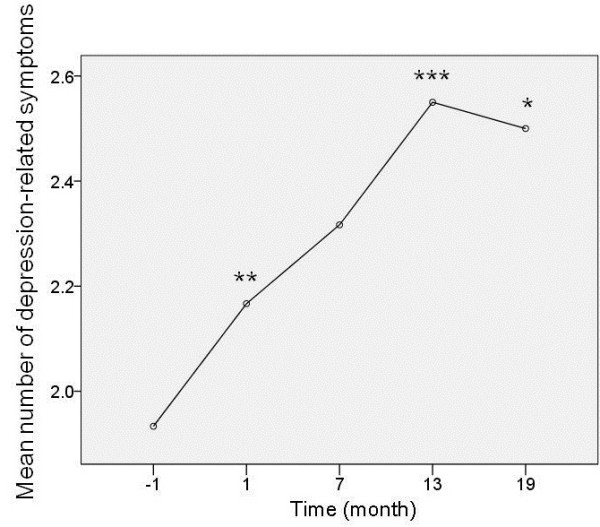
**Change in the number of depression-related symptoms in the fibromyalgia group.** Repeated-measures analysis of variance showed a significant effect of time course in the depression-related symptoms (*F* = 6.68, *P* = 0.001). The Bonferroni *post-hoc* multiple comparisons test revealed significant differences in the number of depression-related symptoms between 1 month before the disaster and 1 month after the disaster (*P* = 0.006)**, between 1 month before the disaster and 13 months after the disaster (*P* <0.0001)***, and between 1 month before the disaster and 19 months after the disaster (*P* = 0.010)*.

Repeated-measures ANOVA showed a significant effect of time course in the depression-related symptoms (*F* = 6.68, *P* = 0.001) (Figure 4). The Bonferroni *post-hoc* multiple comparisons test revealed significant differences in the number of depression-related symptoms between 1 month before the disaster and 1 month after the disaster (*P* = 0.006), between 1 month before the disaster and 13 months after the disaster (*P* <0.0001), and between 1 month before the disaster and 19 months after the disaster (*P* = 0.010). There were no significant correlations between the number of depression-related symptoms and the score of IES-R at any time points (*r* = -0.030, *P* = 0.82 for 1 month after the disaster; *r* = 0.020, *P* = 0.88 for 7 months after the disaster; *r* = -0.067, *P* = 0.61 for 13 months after the disaster; and *r* = -0.096, *P* = 0.46 for 19 months after the disaster).

## Discussion

The present finding of an extremely high IES-R score in the fibromyalgia group 1 month after the disaster, compared with healthy controls or patients with RA as another chronic pain disease, is remarkable. The degree of the score is equivalent to patients with post-traumatic stress disorder (PTSD) [9]. However, the estimated IES-R score in patients with fibromyalgia during usual living was approximately 20. The cutoff value of screening for PTSD is 24/25 in the Japanese version of the IES-R [9], so the baseline value itself in patients with fibromyalgia is extremely high. This finding is consistent with the report on the prevalence of fibromyalgia and PTSD that suggests PTSD is a potential risk factor of fibromyalgia and *vice versa*[11], or with the high prevalence of fibromyalgia after traumatic stress experiences [4,5]. Malt and colleagues found a tendency to over-react to triggers in patients with fibromyalgia using the Eysenck Personality Questionnaire [12]. Lundberg and colleagues found independent personality patterns between fibromyalgia and normal controls triggered by the ability to cope with stress [13]. In fibromyalgia patients, increased harm avoidance and high persistence have been found using the Temperament and Character Inventory [9,11]. In addition, hyperarousal might be intrinsically characteristic in fibromyalgia, considering the subscale score of the IES-R presented here. These characteristics might explain the high IES-R score in the fibromyalgia group.

As mentioned in Results, the mean IES-R score of 7 months after the disaster in the fibromyalgia group had probably already returned to baseline. Accordingly, stress-related symptoms may not have lasted for more than 7 months even in patients with fibromyalgia as well as in patients with RA or in healthy controls. The response to acute stress induced by the great earthquake was thus likely to be settled within 7 months after the disaster. Furthermore, there was no significant difference in change in mean IES-R scores between 1 month and 7 months after the disaster between the groups, suggesting that the amplitude of response to the traumatic event in patients with fibromyalgia may not necessarily be greater than that of other populations.

In contrast to stress-related symptoms, depression-related symptoms in patients with fibromyalgia have been increasing for more than 1 year after the disaster compared with the level before the disaster, despite exclusion of patients with major depression at baseline. This long-lasting worsening of depression-related symptoms may have been a response to chronic stress induced by the fear of radiation due to the nuclear power disaster. These findings suggest that patients with fibromyalgia are vulnerable to chronic stress rather than acute stress.

The disaster consisted of not only a natural disaster but also a manmade disaster; that is, the nuclear power plant accident. Manmade disaster is known to be a risk factor for PTSD [14], and is reported to be associated with high prevalence of fibromyalgia [15]. However, as Tokyo is far from the disaster-stricken areas and the nuclear power plants, all of the subjects in the present study neither received direct physical harm nor were exposed to adverse levels of radiation. Also they did not lose their family member lives. Therefore, whether the disaster was natural or manmade may not sufficiently have influenced the results. Nevertheless, repeated aftershocks of the earthquake and fear of exposure to radiation might have been continuous stress for a while rather than an acute stress. Such continuous stress might have influenced fibromyalgia patients.

In the present study, no significant change in the main symptoms of fibromyalgia represented by the mFS-J or significant change in pain symptoms represented by the WPI was observed. Nevertheless, significant correlations between IES-R and mFS-J and between IES-R and WPI were found. We should mention superficial mismatched results among no significant effects of the time course in the mFS-J, significant changes in the IES-R during the time course, and the linear correlation between IES-R and mFS-J scores or between IES-R and WPI scores. The correlation coefficients between IES-R and mFS-J scores and between IES-R and WPI scores were approximately 0.5 and 0.4, respectively, indicating moderate correlations. Although *P* values of the effect of time course in the mFS-J and WPI were 0.13 and 0.28, respectively, they might become statistically significant in larger samples. Moderate correlation could occasionally be detected in such a situation.

There have so far been two studies on the change in symptoms of fibromyalgia or fibromyalgia-like pain before and after the World Trade Center terrorist attacks on 11 September 2001. Raphael and colleagues reported in a large community sample of women that a cohort initially surveyed for pain and psychiatric symptoms before 11 September were recontacted approximately 6 months after the attacks to assess current symptoms and specific terrorism-related exposures. They concluded that the attacks did not relate to the fibromyalgia-like symptoms [15]. Williams and colleagues found that pain levels of eight fibromyalgia patients in Washington, DC on the day after the attack did not differ significantly from pain levels before the attack [5]. Our result that a significant change in the mFS-J, the severity of fibromyalgia, was not observed is consistent with the previous observations. However, Yunus and colleagues reported that pain severity was influenced by psychological factors in fibromyalgia patients [16]. The moderate but significant relationship between stress-related symptoms and pain in fibromyalgia patients presented here is a new finding, but may be conceptually included in the findings by Yunus and colleagues.

Recently, findings indicating an association between pathophysiology of fibromyalgia and central nervous system dysfunction, such as the default mode network regions [3] and amygdale [17,18], have been accumulating. In PTSD, evidence for disrupted equilibrium between salience and default mode brain networks has been reported [17], as well as dysfunction of amygdala [18]. The vulnerability to traumatic stress in fibromyalgia presented here thus implicates common neural circuitry.

The strengths of the present study are that this is the first describing long-term follow-up of a disease cohort after the disaster, and that the findings represent real clinical practice in Tokyo since the study was performed in a specialized clinic for fibromyalgia that is visited by the largest number of fibromyalgia patients in Japan. A limitation of this study is the imbalance of the number of subjects among groups because this observational study started just after the disaster.

## Conclusions

Although the response to acute stress induced by the great earthquake was likely to be settled within 7 months after the disaster, as indicated by change in the IES-R score, depression-related symptoms have been increasing for more than 1 year after the disaster despite exclusion of patients with major depression at baseline. This long-lasting worsening of depression-related symptoms may have been a response to chronic stress induced by the fear of radiation due to the nuclear power disaster. These findings suggest that patients with fibromyalgia are vulnerable to chronic stress rather than acute stress.

## Abbreviations

ANCOVA: Analysis of covariance; ANOVA: Analysis of variance; IES-R: Impact of Event Scale – Revised; IL: Interleukin; mFS-J: Fibromyalgia symptom scale; PTSD: Post-traumatic stress disorder; RA: Rheumatoid arthritis; TNF: Tumor necrosis factor; WPI: Widespread pain index.

## Competing interests

The authors declare that they have no competing interests.

## Authors’ contributions

CU conceived the hypothesis for the study, participated in data collection, conducted data management, wrote the first draft of the manuscript, and was primarily responsible for the process of manuscript writing. KH, NA and HN conducted statistical analyses and contributed to the study design, analysis and interpretation of data. SO, SA, NY, KN, NA, HN, YY, KI, KN, and TN participated in study design, analysis and interpretation of data. All authors critically reviewed, contributed to, and approved the final manuscript.

## References

[B1] Wolfe F Smythe HA Yunus MB Bennett RM Bombardier C Goldenberg DL Tugwell P Campbell SM Abeles M Clark P The American college of rheumatology 1990 criteria for the classification of fibromyalgia. Report of the multicenter criteria committee Arthritis Rheum 1990 15 160 172 10.1002/art.1780330203 2306288

[B2] Wolfe F Ross K Anderson J Russell IJ Hebert L The prevalence and characteristics of fibromyalgia in the general population Arthritis Rheum 1995 15 19 28 10.1002/art.1780380104 7818567

[B3] Usui C Hatta K Doi N Nakanishi A Nakamura H Nishioka K Arai H Brain perfusion in fibromyalgia patients and its differences between responders and poor responders to gabapentin Arthritis Res Ther 2010 15 R64 10.1186/ar2980 20374641 PMC2888218

[B4] Haviland MG Morton KR Oda K Fraser GE Traumatic experiences, major life stressors, and self-reporting a physician-given fibromyalgia diagnosis Psychiatry Res 2010 15 335 341 10.1016/j.psychres.2009.08.017 20382432 PMC2868959

[B5] Williams DA Brown SC Clauw DJ Gendreau RM Self-reported symptoms before and after September 11 in patients with fibromyalgia JAMA 2003 15 1637 1638 10.1001/jama.289.13.1637 12672730

[B6] Association AP Diagnostic and Statistical Manual of Mental Disorders 1996 4 American Psychiatric Association: Washington, DC

[B7] Aletaha D Neogi T Silman AJ Funovits J Felson DT Bingham CO 3rd Birnbaum NS Burmester GR Bykerk VP Cohen MD Combe B Costenbader KH Dougados M Emery P Ferraccioli G Hazes JM Hobbs K Huizinga TW Kavanaugh A Kay J Kvien TK Laing T Mease P Ménard HA Moreland LW Naden RL Pincus T Smolen JS Stanislawska-Biernat E Symmons D Rheumatoid arthritis classification criteria: an American college of rheumatology/European league against rheumatism collaborative initiative Ann Rheum Dis 2010 15 1580 1588 10.1136/ard.2010.138461 20699241

[B8] Weiss DS The Impact of Event Scale – Revised 2004 2 New York: The Guilford Press

[B9] Asukai N Kato H Kawamura N Kim Y Yamamoto K Kishimoto J Miyake Y Nishizono-Maher A Reliability and validity of the Japanese-language version of the impact of event scale-revised (IES-R-J): four studies of different traumatic events J Nerv Ment Dis 2002 15 175 182 10.1097/00005053-200203000-00006 11923652

[B10] Usui C Hatta K Aratani S Yagishita N Nishioka K Kanazawa T Itoh K Yamano Y Nakamura H Nakajima T The Japanese version of the modified ACR preliminary diagnostic criteria for fibromyalgia and the fibromyalgia symptom scale: reliability and validity Mod Rheumatol 2013 15 846 850 10.1007/s10165-012-0759-x 23001748

[B11] Hauser W Galek A Erbsloh-Moller B Kollner V Kuhn-Becker H Langhorst J Petermann F Prothmann U Winkelmann A Schmutzer G Brähler E Glaesmer H Posttraumatic stress disorder in fibromyalgia syndrome: prevalence, temporal relationship between posttraumatic stress and fibromyalgia symptoms, and impact on clinical outcome Pain 2013 15 1216 1223 10.1016/j.pain.2013.03.034 23685006

[B12] Malt EA Olafsson S Lund A Ursin H Factors explaining variance in perceived pain in women with fibromyalgia BMC Musculoskelet Disord 2002 15 12 10.1186/1471-2474-3-12 12019032 PMC113754

[B13] Lundberg G Anderberg U Gerdle B Personality features in female fibromyalgia syndrome J Musculoskelet Pain 2009 15 117 130 10.1080/10582450902820531

[B14] Dirkzwager AJ Grievink LV van der Velden PG Yzermans CJ Risk factors for psychological and physical health problems after a man-made disaster. Prospective study Br J Psychiatry 2006 15 144 149 10.1192/bjp.bp.105.017855 16880484

[B15] Raphael KG Natelson BH Janal MN Nayak S A community-based survey of fibromyalgia-like pain complaints following the world trade center terrorist attacks Pain 2002 15 131 139 10.1016/S0304-3959(02)00273-7 12435466

[B16] Yunus MB Ahles TA Aldag JC Masi AT Relationship of clinical features with psychological status in primary fibromyalgia Arthritis Rheum 1991 15 15 21 10.1002/art.1780340104 1984776

[B17] Sripada RK King AP Welsh RC Garfinkel SN Wang X Sripada CS Liberzon I Neural dysregulation in posttraumatic stress disorder: evidence for disrupted equilibrium between salience and default mode brain networks Psychosom Med 2012 15 904 911 10.1097/PSY.0b013e318273bf33 23115342 PMC3498527

[B18] Mahan AL Ressler KJ Fear conditioning, synaptic plasticity and the amygdala: implications for posttraumatic stress disorder Trends Neurosci 2012 15 24 35 10.1016/j.tins.2011.06.007 21798604 PMC3206195

